# Cutaneous lupus erythematosus disease assessment: Highlighting CLE outcome measures

**DOI:** 10.3389/fmed.2022.968469

**Published:** 2022-10-14

**Authors:** Rebecca G. Gaffney, Victoria P. Werth, Joseph F. Merola

**Affiliations:** ^1^Harvard Combined Dermatology Program, Boston, MA, United States; ^2^Department of Dermatology, University of Pennsylvania, Philadelphia, PA, United States; ^3^Corporal Michael J. Crescenz Veteran's Administration Medical Center (VAMC), Philadelphia, PA, United States; ^4^Division of Rheumatology, Department of Dermatology and Medicine, Brigham and Women's Hospital, Boston, MA, United States

**Keywords:** cutaneous lupus disease area and severity index, Cutaneous lupus erythematosus (CLE), investigator global assessment, Systemic Lupus Erythematosus Disease Activity Index (SLEDAI), Skindex-29, Dermatology Life Quality Index (DLQI), SF-36

## Introduction

Cutaneous lupus erythematosus (CLE) is an autoimmune skin disease that can occur with or without systemic lupus erythematosus (SLE). This potentially disfiguring disease can have significant impact on patient's quality of life (QoL) and is often refractory to many first- and second-line therapies. Despite the need for new treatments in CLE, patients are often excluded from clinical trials with SLE due to disease heterogeneity and previously difficult to measure disease activity and QoL. Standardized outcome measures for CLE are essential for trial design and regulatory approval of novel treatments. In this review, we aim to explore and highlight the various outcome measures for physician reported outcomes and patient reported outcomes for CLE.

CLE is an autoimmune skin disease that can occur with or without features of SLE. Even skin-limited disease can have a significant impact on patient's QoL and patients are often refractory to standard topical treatment and antimalarials. Despite this, there have been no skin-directed therapies approved by US Food and Drug Administration (FDA) in the past 50 years, and only two new biologics for SLE during this time frame (1, 2). Patients with CLE are often excluded from clinical trials for SLE, likely due to disease heterogeneity and previously difficult to measure disease activity and QoL in these patients. More clinical trials focusing on CLE are emerging and CLE specific outcome measures are important in identifying promising medications in this potentially disfiguring disease.

Owing to the challenges with outcomes research in this heterogeneous disease, there is currently a lack of standardized outcome measures in CLE to be used in clinical trials. This represents a barrier to trial design, problematic heterogeneity across studies, and a regulatory hurdle to approval of much-needed novel drugs. Our group recently published a multistage literature review of CLE and SLE studies to develop a working core outcome set (COS) for CLE to be used in clinical trials as an interim guide until standardized outcomes are established (3). Proposed core domains include skin-specific disease activity and damage, investigator global assessment of disease activity, symptoms (encompassing itch, pain, and photosensitivity), health related quality of life, and patient global assessment of disease activity. In this review, we aim to highlight our recommended outcome measures for each core domain and summarize other various physician reported outcomes and patient reported outcomes that have been used for CLE.

## Physician reported outcomes

### Skin-specific instruments

#### Cutaneous lupus disease area and severity index

The CLASI was developed by an international group of experts in dermato-rheumatology who met multiple times to discuss and review descriptors, as a responsive CLE disease measurement tool to be used in clinical trials. Patients were also interviewed to make sure the CLASI captured what was important to them. Subsequent qualitative studies confirmed the items chose for the CLASI reflected concerns to patients (4). It was designed to capture various CLE subtypes, but excluded more rare entities like lupus panniculitis and bullous lupus. The CLASI has two scores: activity and damage. Each anatomic location is scored (from scalp to toes), with highly photo-exposed areas listed separately in addition to sections focusing on mucous membrane involvement and alopecia. For the activity score, points are given for mucous membrane lesions, recent hair loss, diffuse hair loss attributed to active SLE, inflammatory scalp alopecia, as well as the presence of erythema and scale in multiple different body surface areas to allow for determining the extent of disease without relying on Body Surface Area (BSA), which may be quite low, even in extensive active disease. Separate composite scores for activity are calculated by simply summing the individual component scores. Disease activity is scored to a maximum of 70 points. For damage score, points are given for presence of dyspigmentation and scarring including scarring alopecia to a maximum of 80 points. Dyspigmentation score is doubled when it has been present for more than 12 months (1, 5).

The CLASI has shown excellent content validity, construct validity, and inter/intra-rater reliability in multiple studies and has been validated for use in the pediatric population (1, 6). Additionally, the CLASI has shown to correlate with QoL. A severity and responsive analysis showed that higher numerical score indicated more severe disease. Therefore, a reduction in CLASI score corresponds to a reduction in disease activity which makes it an excellent organ-specific outcome measure to use in clinical trials (7). Our prior literature review showed that the CLASI was used in 54.5% (*n* = 18) of CLE and SLE randomized control trials that evaluated skin with a skin-specific outcome measure and 66.7% of CLE and SLE studies published in PubMed or ClinicalTrials.gov since it was developed and validated. The CLASI-A and CLASI-D were therefore recommended instruments for the core domains of skin-specific disease activity and skin-specific disease damage in our proposed working core outcome set (3).

Unlike the Psoriasis Area and Severity Index (PASI), which is the gold standard to measure severity and extent of psoriasis, the CLASI does not take surface area into account in scoring (8). Affected areas are weighed equally regardless of surface area or number of lesions, but scores are assigned based on the most severe representative lesion in each anatomic area. However, in CLE, surface area is often small and may not reflect true disease severity. Patients may have numerous small lesions that significantly impact QoL without adding up to a large BSA. Like the PASI, the CLASI uses erythema as a hallmark of disease activity by reflecting the hyperemia that accompanies inflammation. Since erythema can be transient or reflect underlying telangiectasia, using the CLASI requires training to be able to accurately score patients (1). Finally, in the original validation studies, it was found that the CLASI takes an average duration of 5.25 min to conduct (ranging from <1–11 min). There was no significant variation over time as experience with the instrument increased (5).

The CLASI works for most subsets of CLE, with the exception of lupus panniculitis and tumid LE if there is no erythema, which is quite rare. The activity of lupus panniculitis is difficult to assess and thus is not included in the CLASI except as relates to the lipoatrophy from resolved panniculitic activity. The RCLASI is partially validated and has demonstrated good inter/intra-rater reliability, but its practicality is limited by the extensive nature of this instrument. The less user-friendly nature of the RCLASI has drawn into question its feasibility for use in clinical trials (9).

#### Cutaneous lupus activity-investigator global assessment

The FDA previously released a document for the development of drugs for SLE with emphasis on treatment measurement of disease activity and damage and are now encouraging disease-specific global assessment tools for many inflammatory skin disorders (1, 10). Per our prior literature review performed, there was no standardized IGA for CLE (3). Thus, the CLA-IGA was recently developed by experts in dermato-rheumatology to fill this unmet need. It is currently undergoing reliability studies and therefore its validity and applicability is not yet determined.

Scoring is based on severity of morphologic features of CLE. Like other IGA instruments, it consists of a 5-point scale (0 = clear, 1 = almost clear, 2 = mild, 3 = moderate, and 4 = severe) that evaluates the severity of the CLE disease activity. Scoring is based on the severity of the morphologic features averaged across all body lesions. Morphologic features include erythema, scale, edema/infiltration, the extent of follicular plugging/follicular hyperkeratosis of the scalp, and secondary changes of CLE plaques such as presence of vesicles, erosion, and crusting.

Item generation across the breadth of CLE subsets was derived from a large international consensus exercise and was subsequently drafted by experts in connective tissue dermatology. Its content validity was further developed by involvement by a larger panel of dermato-rheumatology experts over several rounds of input to refine morphologic features and review content descriptors. Like the CLASI, it uses erythema as a driver for final score and morphologic characteristics were selected to reflect severity of CLE disease activity and be sensitive to change over time.

The CLA-IGA offers a CLE-specific global assessment tool that can provide a snapshot of overall disease activity and is highly feasible to perform. Because an IGA is a more global assessment with an ordinal scale, it also allows for disease severity to be readily and easily interpretable by clinicians and patients alike. It offers complementary data to the CLASI, focusing on lesion morphology activity severity, without the need for extent of disease considerations. This may be particularly relevant in the most common subsets of CLE where BSA extent is often limited but still carries high burden on patients. It may additionally be considered for studies with lower BSA and/or for assessing target lesions. Analogies may be drawn between the common use of concurrent PASI and psoriasis IGA in the conduct of psoriasis trials. Given the heterogeneity of CLE presentation, often with more than one subtype in the same patient, assignment of features such as specific level of erythema or more subtle changes in activity may be challenging to capture with the CLA-IGA where regional variation may exist. Nevertheless, global assessment tools are supported and encouraged by the FDA and the CLA-IGA offers a complement to the CLASI in clinical trials. We therefore recommend the CLA-IGA as a possible endpoint for CLE pending results of validation studies (3).

### SLE instruments that measure skin involvement

The Systemic Lupus Erythematosus Disease Activity Index (SLEDAI) is a global index that measures disease activity through 24 questions regarding clinical manifestations of SLE (physical findings and laboratory values) that are weighted by type of manifestation, not severity of the manifestation. For example, preferential weighting is given to vasculitis, central nervous system involvement, and active renal disease. The maximum score achievable is 105, but even patients with very active disease rarely exceed a score of 20. “Inflammatory-type rash,” “alopecia,” and “oral or nasal ulcers” are the only representations of skin findings in this tool. Additionally, because the SLEDAI only measures the presence or absence of features, skin disease needs be completely resolved to indicate improvement, making it insensitive to incomplete resolution of changes. The SLEDAI-2K was developed as a modification to the SLEDAI to reflect persistent, active disease in scoring and has been validated against the original SLEDAI as a predictor of mortality and measure of disease activity (11, 12).

Similar tools to the SLEDAI/SLEDAI 2K are the Lupus Activity Criteria Count (LACC), the British Isles Lupus Assessment Group (BILAG), and the Systemic Lupus Collaborative Clinics/American College of Rheumatology Damage Index for SLE (SLICC/ACR Damage Index for SLE) which also only document the presence or absence of CLE manifestations, and are therefore not adequate tools to evaluate CLE disease activity (1, 12, 13).

### Patient reported outcomes

#### Health related quality of life

The Dermatology Life Quality Index (DLQI) is a widely-used dermatology-specific questionnaire consisting of ten questions about the previous 1 week. The total DLQI ranges between 0 (no impairment) and 30 (maximum impairment). The ten questions in the DLQI can be subdivided into six domains that relate to different aspects as follows: symptoms and feelings, daily activities, leisure, work/school, personal relationships, and treatment (14). There is just one question related to the impact on emotional QoL, and emotions are greatly impacted in CLE (14). We found just one validation study for the Brazilian version of the DLQI for CLE (3, 15).

The Skindex-29 is a validated measure of the effects of skin disease on QoL. There are 29 items that form three domains: symptoms, emotions, and functioning. The symptoms subscale measures pain, itch, burning, or sensitivity. The emotional subscale measures depression, anxiety, embarrassment, or anger. The functioning subscale evaluates changes to daily life, such as work, sleep, or relationships with others. In the Skindex+3, there is a fourth subscale that assesses CLE-specific issues such as photosensitivity and alopecia. Patients are asked to assess how often (never, rarely, sometimes, often, all the time) they experience a given effect and scores are assigned to each question. Domain scores and overall score are expressed on a 100 point scale with higher numbers indicating worse QoL (16).

The CLE-QoL was recently developed with input from patients with CLE. It combines the Skindex-29+3 with four questions from the vitiligo-specific quality of life (VitiQoL) instrument. The four additional questions correspond to an additional subscale in body image/cosmetic issues. It has shown strong reliability and structural and convergent validity in a single validation study, and future studies will determine if additional questions meaningfully improve the capture of QoL features (17).

Interestingly, a study was performed in patients with psoriasis and eczema (*n* = 28) to compare the DLQI and Skindex-29. Interviews on content and format of both tools showed that participants preferred the Skindex-29 for ease of understanding and incorporation of various emotions. Patients were overall satisfied with format and length of both tools (18).

Other generic QoL indices identified in our literature review include the EQ-5D and the Short Form Health Survey (SF-36), which have not been validated in CLE (3).

#### Patient global assessment of disease

The PtGA is an instrument that allows for a subjective overall evaluation of disease severity from the patient's perspective. It is a widely use PRO across multiple diseases, including skin-specific entities. There are multiple scoring systems, but most PtGA instruments use an ordinal scale to rate severity of disease on a 5-point scale where 0 = clear, 1 = almost clear, 2 = mild, 3 = moderate and 4 = severe. A 10-point linear VAS scale has been used for a number of studies. Despite feasibility of use, there is currently no validated PtGA for disease activity in CLE (3).

#### Patient reported symptoms

While the Skindex-29+3 and CLE-QoL both include questions about itch, pain, and photosensitivity, there are no CLE-specific measurements found dedicated to these symptoms (16, 17).

The 12-Item Pruritus Scale (12-PSS) has been shown to be a valid and reliable tool to assess generic dermatologic itch. It is a one-page instrument that consists of 12 items to assess different aspects of pruritus. Though it was not originally developed for patients with CLE, severity bands were later defined for CLE (19).

Commonly used and practical scales in clinical trials, though not formally validated in CLE, are the pain and pruritus Visual Analog Scales (VAS) and Numeric Rating Scales (NRS). For the VAS, patients are asked to mark a position along a 10 cm long line that corresponds to a single question about pain or itch and severity is assigned based on length. Similarly, for the NRS, patients are asked to rate their symptoms on a defined scale between 0 and 10.

In our proposed core outcome set for PROs, we were unable to recommend one clearly superior instrument due to lack of validation data and the vast number of instruments identified. However, suitable instruments include the CLE-QoL, Skindex29+3, DLQI, SF-36, and EQ-5D for HRQoL domain and the 12-PSS, CLE-QoL, Skindex29+3, DLQI, itch VAS/NRS, and pain VAS/NRS for the symptoms domain. Given the lack of CLE-specific PtGAs, there is no specific outcome measure that could be recommended for the patient global assessment domain (3).

A summary of commonly used outcome measurements can be found in Table 1.

**Table 1:** Lupus outcome measurements.

**Outcome measurements**	
**Physician reported outcomes**	**Patient reported outcomes**
Skin specific instruments	The Dermatology Life Quality Index (DLQI)
Cutaneous Lupus disease Area and Disease Severity Index (CLASI)	Skindex-29
Cutaneous Lupus Activity-Investigator Global Assessment (CLA-IGA)	Cutaneous Lupus Erythematosus-Quality of Life (CLE-QoL)
	Patient Global Assessment of Disease (PtGAs)
SLE instruments that measure skin involvement	12-Item Pruritus Scale (12-PSS)
The Systemic Lupus Erythematosus Disease Activity Index (SLEDAI)	Visual Analog Scales (VAS)
	Numeric Rating Scales (NRS)

## Conclusion

CLE represents a set of conditions with heterogeneous presentation, variably associated with underlying SLE. The heterogeneity in both CLE presentation and CLE outcome measures has previously hindered trial design and drug development. To help overcome this barrier, we recently developed a working core outcome set for CLE and our recommended outcome measures for each core domain are reviewed above. This COS can serve as an interim guide for upcoming CLE trials but large-scale consensus exercises are ideal to develop standardized outcome measures. The previous lack of focus on skin outcomes in trials was significantly improved by the CLASI, and validation studies for the FDA requested CLA-IGA are underway. This review identifies a paucity of validated CLE-specific patient reported outcomes, particularly a PtGA. The CLE-QoL is a newer and promising instrument that should be included in future studies to further evaluate its validity and responsiveness.

In this review, we identify the current CLE outcome measures and highlight unmet needs that will hopefully inform the agenda for future studies to allow a regulatory pathway forward to develop novel drugs for CLE.

## Author contributions

RG, JM, and VW made substantial contributions to the conception or design of the work, or the acquisition, analysis, or interpretation of data for the work, drafting the work or revising it critically for important intellectual content, provided approval for publication of the content, and agree to be accountable for all aspects of the work in ensuring that questions related to the accuracy or integrity of any part of the work are appropriately investigated and resolved. All authors contributed to the article and approved the submitted version.

## Conflict of interest

The authors declare that the research was conducted in the absence of any commercial or financial relationships that could be construed as a potential conflict of interest.

## Publisher's note

All claims expressed in this article are solely those of the authors and do not necessarily represent those of their affiliated organizations, or those of the publisher, the editors and the reviewers. Any product that may be evaluated in this article, or claim that may be made by its manufacturer, is not guaranteed or endorsed by the publisher.
